# Predicting binding sites of hydrolase-inhibitor complexes by combining several methods

**DOI:** 10.1186/1471-2105-5-205

**Published:** 2004-12-17

**Authors:** Taner Z Sen, Andrzej Kloczkowski, Robert L Jernigan, Changhui Yan, Vasant Honavar, Kai-Ming Ho, Cai-Zhuang Wang, Yungok Ihm, Haibo Cao, Xun Gu, Drena Dobbs

**Affiliations:** 1 L.H. Baker Center for Bioinformatics and Biological Statistics, Iowa State University, Ames, IA 50011, USA; 2 Department of Biochemistry, Biophysics, and Molecular Biology, Iowa State University, Ames, IA 50011, USA; 3 Department of Computer Science, Iowa State University, Ames, IA 50011, USA; 4 Bioinformatics and Computational Biology Program, Iowa State University, Ames, IA 50011, USA; 5 Department of Physics and Astronomy, Iowa State University, Ames, IA 50011, USA; 6 Department of Genetics, Development and Cell Biology, Iowa State University, Ames, IA 50011, USA

## Abstract

**Background:**

Protein-protein interactions play a critical role in protein function. Completion of many genomes is being followed rapidly by major efforts to identify interacting protein pairs experimentally in order to decipher the networks of interacting, coordinated-in-action proteins. Identification of protein-protein interaction sites and detection of specific amino acids that contribute to the specificity and the strength of protein interactions is an important problem with broad applications ranging from rational drug design to the analysis of metabolic and signal transduction networks.

**Results:**

In order to increase the power of predictive methods for protein-protein interaction sites, we have developed a consensus methodology for combining four different methods. These approaches include: data mining using Support Vector Machines, threading through protein structures, prediction of conserved residues on the protein surface by analysis of phylogenetic trees, and the Conservatism of Conservatism method of Mirny and Shakhnovich. Results obtained on a dataset of hydrolase-inhibitor complexes demonstrate that the combination of all four methods yield improved predictions over the individual methods.

**Conclusions:**

We developed a consensus method for predicting protein-protein interface residues by combining sequence and structure-based methods. The success of our consensus approach suggests that similar methodologies can be developed to improve prediction accuracies for other bioinformatic problems.

## Background

Protein-protein interactions play a critical role in protein function. Completion of many genomes is being followed rapidly by major efforts to identify experimentally interacting protein pairs in order to decipher the networks of interacting, coordinated-in-action proteins. Identification of protein-protein interaction sites and detection of specific residues that contribute to the specificity and strength of protein interactions is an important problem [1-3] with broad applications ranging from rational drug design to the analysis of metabolic and signal transduction networks. Experimental detection of residues on protein-protein interaction surfaces can come either from determination of the structure of protein-protein complexes or from various functional assays. The ability to predict interface residues at protein binding sites using computational methods can be used to guide the design of such functional experiments and to enhance gene annotations by identifying specific protein interaction domains within genes at a finer level of detail than is currently possible.

Computational efforts to identify protein interaction surfaces [4-6] have been limited to date, and are needed because experimental determinations of protein structures and protein-protein complexes, lag behind the numbers of protein sequences. In particular, computational methods for identifying residues that participate in protein-protein interactions can be expected to assume an increasingly important role [4,5]. Based on the different characteristics of known protein-protein interaction sites [7], several methods have been proposed for predicting interface residues using a combination of sequence and structural information. These include methods based on the presence of "proline brackets"[8], patch analysis using a 6-parameter scoring function [9,10], analysis of the hydrophobicity distribution around a target residue [7,11], multiple sequence alignments [12-14], structure-based multimeric threading [15], and analysis of amino acid characteristics of spatial neighbors to a target residue using neural networks [16,17]. Our recent work has focused on prediction of interface residues by utilizing analyses of sequence neighbors to a target residue using SVM and Bayesian classifiers [2,3].

There is an acute need for multi-faceted approaches that utilize available databases of protein sequences, structures, protein complexes, phylogenies, as well as other sources of information for the data-driven discovery of sequence and structural correlates of protein-protein interactions [4,5]. By exploiting available databases of protein complexes, the data-driven discovery of sequence and structural correlates for protein-protein interactions offers a potentially powerful approach.

## Results and discussion

Here we are using a dataset of 7 hydrolase complexes from the PDB, together with their sequence homologs. The application of our consensus method to other types of complexes, *e.g*. antibody-antigen complexes is currently under study and will be published later. It should be noted, however, that prediction of binding sites for other types of protein complexes, especially those involved in cell signaling, is likely to be more difficult than for the hydrolase-inhibitor complexes.

**Figure 1:**
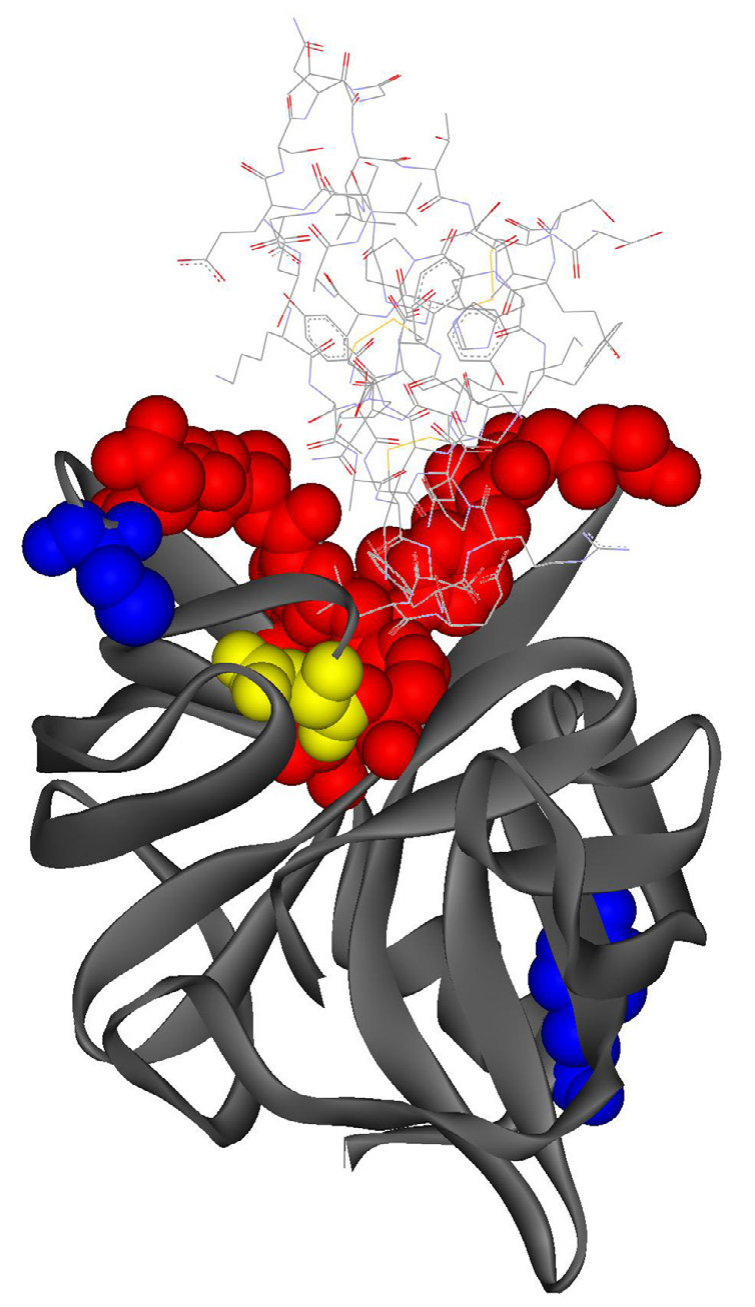
Interface residues predictions mapped on the three dimensional structure of Proteinase B from *Streptomyces griseus *(3sgb). The target protein is shown in ribbons and atomic spheres; the inhibitor partner is shown at the top in faint wire frame. The residues are color coded as: red = true positives (TP), gray = true negatives (TN), yellow = false negatives (FN), and blue = false positives (FP). Red, yellow, and blue residues are shown in spacefill representation. Note that the actual interface residues extracted from the PDB structure include the red (TP) and yellow (FN) residues. Red and gray residues represent correct predictions of interface and non-interface residues (14 TP+ 210 TN = 224 correct predictions); yellow and blue residues represent incorrect predictions (1 FN + 2 FP= 3)

Figure 1 shows an example of the consensus method prediction mapped on the structure of proteinase B from *S. griseus *in a complex with turkey ovomucoid inhibitor (PDB 3sgb [18]). The inhibitor (3sgb_I) is shown at the top in wire frame and the proteinase B chain (3sgb_E), is shown at bottom. Actual interface residues in the proteinase B chain, i.e., amino acids that form the binding site between proteinase B and the inhibitor, were extracted from the PDB structure (see Materials and Methods). Predicted interface and non-interface residues, identified by the consensus method, are shown as color coded atoms as follows: Red spheres = true positives (TP), actual interface residues that are predicted as such; Gray strands = true negatives (TN), non-interface residues that are predicted as such; Yellow spheres = false negatives (FN), interface residues that are misclassified as non-interface residues; Blue spheres = false positives (FP), non-interface residues that are misclassified as interface residues. Note that the binding site in proteinase B is strongly indicated, with 14 out of 15 interface residues correctly classified, along with 2 false positives.

**Figure 2:**
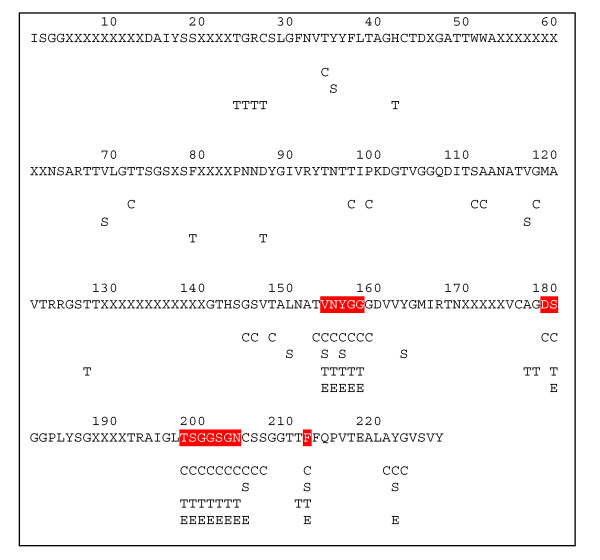
Comparison of individual methods for interface residue prediction with the consensus method. Results are shown for Proteinase B from *Streptomyces Griseus *(3sgb_E), the same protein shown in Figure 1. Actual interfaces are highlighted in red. Interface residues predicted by each of five different methods are indicated as follows: P = Phylogeny (none predicted for this protein), C = Conservatism of Conservatism; S = Support Vector Machine; T = Threading; and E = Consensus. Amino acid residues present in the protein sequence, but not included in the PDB structure file, are indicated by "X"s in the sequence.

The primary amino acid sequence for proteinase B chain and the interface residue prediction results for the four individual methods and the consensus method are shown in Figure 2. Actual interface residues are identified highlighted in red. The five lines below the amino acid sequence show the locations of interface residues predicted by the different methods (described in detail below): P = Phylogeny; C = Conservatism of Conservatism (CoC); S = Data mining by SVM; T = Threading; E = Consensus. Similar Figures for each protein studied in this work are provided in Supplementary Materials [see Additional files 1, 2, 3, 4, 5, and 6].

The prediction results for all methods are shown in Table 1 and Table 2. Table 1 shows a complete summary of the classification performance on the proteinase B chain for all 5 methods including the overall Sensitivity (Sen) and Specificity (Spec); Sensitivity (Sen+) and Specificity (Spec+) for interface residues (the "positive" class); and Correlation Coefficient (see Materials and Methods for definitions of these performance parameters). Table 2 shows the overall average performance results for all seven protein complexes studied in this work. Two kinds of averages are considered: the numerical average over each of 7 proteins in the dataset, i.e., the average on a "per protein" basis (<...>_p_); and the average over the total number of residues, i.e., the average on a "per residue" basis (<...>_r_).

**Table 1:** Classification results for Proteinase B from *S. griseus *(3sgb_E). TP is the number of true positive; TN is the number of true negatives; FP is the number of false positives, and FN is the number of false negatives. Overall sensitivity, overall specificity, sensitivity+, specificity+, and correlation coefficient are defined in the text.

**3SGBE**	**TP #**	**TN #**	**FP #**	**FN #**	**Overall Sen**	**Overall Spe**	**Sen+**	**Spe+**	**CC**
**Phylog.**	0	212	0	15	0.94	0.91*	0	-	0*
**COC**	15	194	18	0	0.92	0.96	1	0.45	0.64
**SVM**	3	205	7	12	0.92	0.90	0.20	0.30	0.20
**Thread.**	14	201	11	1	0.95	0.97	0.93	0.56	0.70
**Cons.**	14	210	2	1	0.99	0.99	0.94	0.88	0.90

**Table 2:** Overall Classification Performance Results Averaged over 7 Proteins. Average results for Sensitivity+, Specificity+, overall Sensitivity, overall Specificity, and Correlation Coefficient averaged over the 7 proteins in the dataset. <>_p_denotes averaging over the total number of proteins, <>_r_denotes averaging over the total number of residues.

**Method**	**<Sen+>_p_**	**<Spe+>_p_**	**<Spe>_p_**	**<Spe>_r_**	**<Sen>_p_**	**<Sen>_r_**	**<Cor>_p_**	**<Cor>_r_**
**Phylog.**	0.39	0.71	0.90	0.89	0.91	0.89	0.43	0.37
**COC**	0.71	0.31	0.89	0.88	0.81	0.80	0.38	0.37
**SVM**	0.51	0.41	0.89	0.88	0.88	0.88	0.39	0.37
**Thread.**	0.59	0.57	0.91	0.89	0.92	0.91	0.53	0.48
**Cons.**	0.70	0.56	0.92	0.91	0.90	0.89	0.56	0.55

### Sequence and structure conservation

Amino acid sequences are conserved for many different reasons related to the structure and function of proteins: for stability [19,20], enzyme active sites, subunit interfaces, facilitation of an essential motion (hinges), and binding sites. Developing methods to identify the reason for conservation of individual highly conserved residues is a difficult problem. This is one of the reasons that a combination of approaches may be more likely to permit identification of residues that participate in protein-protein interactions. Even identifying the conserved residues themselves is not completely straightforward, and as will be seen, different approaches will indicate the same residue being conserved to different extents. In this study, we take advantage of this by using several methods to identify sequence and structure conservation. Here we use two principal methods for this purpose, one based on phylogeny to identify sequence conservation and one based on Conservatism of Conservatism [21] to identify structure conservation. These two methods often identify different residues as being conserved.

### Phylogeny

To identify protein residues that are conserved – perhaps due to their functional role in forming specific protein-protein interactions – we use ClustalX [22] multiple sequence alignments of protein sequences to generate phylogenetic trees (see Materials and Methods). Conserved residues are defined as those that are identical at a given position in more than 85% alignments, i.e., only 15% substitutions or gaps were allowed. This 85% cutoff value is found to give optimal results (data not shown). Because phylogenetic trees of closely related sequences result in many residues that satisfy this condition (due to the high conservation of sequences, apparently important for protein folding, located in the protein core) we filter the results to focus on surface residues by removing conserved residues residing inside the protein core, i.e., having low solvent accessibility (see Materials and Methods).

As shown in Figure 2, the phylogenetic method does not classify any of the amino acids in proteinase B chain (3sgb_E) as interface residues, i.e., TP = 0 and FP = 0. Thus, for the phylogenetic method prediction, the correlation coefficient (CC), which can range from -1 to +1, converges to zero, whereas overall specificity converges to 0.905. The latter misleading statistic is due to the large number of negative examples (non-interface residues), which are correctly classified. In cases such as this (with unbalanced numbers of positive and negative examples), *sensitivity*+ and *specificity*+ measures are especially useful because they more clearly reflect the ability of a method to detect "positive" interface residues. (See the Methods section for definition and further discussion of performance measures). Note that even though Figure 2 shows that the phylogenetic method does not identify any interface residues in this particular example, the results summarized in Table 1 for all seven proteins demonstrate that the ability of the phylogenetic method to correctly predict non-interface residues (reflected in the high overall sensitivity and specificity values), and in combination with other methods, to lead to significantly improved predictions.

### Conservatism of conservatism

To detect structurally conserved residues that are possible binding sites we have used the Conservatism of Conservatism method (CoC) developed by Mirny and Shakhnovich[21] We use structural alignments generated by FSSP (fold classification based on structure-structure alignment of proteins) developed by Holm and Sander [23]) to identify protein families with folds similar to that of the each of the 7 proteins. For each family, HSSP [24] (homology-derived secondary structure of proteins) alignments are used to calculate the sequence entropy at each position of the alignment. The HSSP profile is based on the multiple alignment of a sequence and its potential structural homologues [25]. The structural alignment generated by FSSP is used to calculate the value of CoC (see Materials and Methods). Each residue in the protein chain was ranked according to its CoC value at a given position in the sequence. The top 75% of total residues ranked according to their CoC values are defined as conserved. We filter the results of the CoC ranking by removing all structurally conserved residues located inside the protein core by only choosing the residues that have a relative accessibility of at least 25 as calculated by DSSP [26] (dictionary of protein secondary structure). Interface residues in proteinase B predicted by this method are indicated by a "C" in Figure 2. The overall performance of the CoC method is summarized in the second row of Tables 1 and 2. Although the correlation coefficient of the COC method is in the same range of those obtained by phylogeny and support vector machines, 0.37, the sensitivity+ value, 0.71, is surpassed only by the consensus value. Therefore, a larger fraction of interface residues is predicted by CoC than the other three methods. However, the CoC method alone is not sufficient to successfully predict binding sites, and combining this method with other prediction techniques in the consensus method gives improved results (Tables 1 and 2).

### Data mining for binding residues

We have generated a support vector machine (SVM) classifier to determine whether or not a surface residue is located in the interaction site using information about the sequence neighbors of a target residue. An 11-residue window consisting of the residue and its 10 sequence neighbors (5 on each side) is chosen empirically. Each amino acid in the 11 residue window is represented using 20 values obtained from the HSSP profile of the sequence. Each target residue is therefore associated with a 220 (11 × 20) element vector. The SVM learning algorithm is given a set of labeled examples of the form (X, Y) where X is the 220 element vector representing a target residue and Y is its corresponding class label, either interface or non-interface residue. The SVM algorithm generates a classifier which takes as input a 220 element vector that encodes a target residue to be classified and outputs a class label. Our previous study [2] reported results for classifiers constructed using a combined set of 115 proteins belonging to six different categories of complexes: antibody-antigen, protease-inhibitor, enzyme complexes, large protease complexes, G-proteins, cell cycle signaling proteins, signal transduction, and miscellaneous. In another study [3], we trained separate classifiers for each major category of complexes (e.g., protease-inhibitor complexes). In the case of protease-inhibitor complexes, leave-one-out experiments were performed on a set of 19 proteins. In each experiment, an SVM classifier was trained using a set of surface residues, labeled as interface or non-interface, from 18 of the 19 proteins. The resulting classifier was used to classify the surface residues of the remaining target protein into interface residue and non-interface residue categories. The interface residues obtained for 3sgb_E are reproduced in Figure 2 and marked by "S". The performance of the SVM classifier for the current test set of complexes is summarized in Tables 1 and 2. The results show that SVM yields relatively high sensitivity+ (0.51) and specificity+ (0.41).

### Threading of sequences through structures of interface surfaces

Structural threading was performed for the set of 7 protein complexes using a recently developed threading algorithm [27], which was first used in the CASP5 [28] competition. For each complex structure, we first extract the interfacial region, essentially as described earlier. Residue-residue contacts in the interfacial region are described with contact matrices. The total energy in this threading method is the sum of all pair-wise contact energies for the conformation. Detailed residue-level contact potentials were obtained from the Li, Tang and Wingreen [29] parameterization of the Miyazawa and Jernigan [30] matrix. We represent a protein sequence vector **s **by the hydrophobicity values of its amino acids *h*_*i *_obtained in this factorization and protein structure by the contact matrix Γ. The problem of finding the best alignment of a query sequence **s **with a structure having contact matrix Γ is to find the transformation from **s **to **s' **that optimizes the energy function. The optimum **s' **is the dominant eigenvector **v_0 _**of the contact matrix Γ. There is a strong correlation between a protein sequence and the dominant eigenvector of its native structure's contact matrix. Here the transformation we seek is obtained by maximizing the correlation between **s' **and **v_0_**. This is an alignment problem, and a dynamic programming method from sequence alignment has been adapted to solve this problem [27].

For each sequence, threading is performed against structures in our template database and alignment results used only when the score exceeds a length-dependent threshold. From the alignments, residues involved in contacts at the interface are identified using a scale based on the number of times a particular residue is indicated and the strength of the threading score. The predicted binding sites for 3sgb_E by the threading method are marked in Figure 1 by "T" and the prediction results are summarized in Tables 1 and 2. The threading-based approach is somewhat more successful than other methods based on its sensitivity+, selectivity+, and correlation coefficient values, but still not as good as the performance obtained by combining it with methods in the consensus approach.

### Consensus method for predicting protein binding sites

Based on the results from the predictions with the four independent methods, we have developed a simple consensus method to obtain a better prediction. In the consensus method results presented here, an amino acid is considered to be an interface residue if any of the following conditions are met:

i) at least three independent methods classify it as an "interface residue"

ii) any two methods (except the Phylogeny-Threading pair) predict it

For this set of proteins, the parameters for combining results in the consensus method have been empirically determined without a systematic comparison of the strengths and weaknesses of each method. We employ this simple approach because it provides demonstrable improvement in prediction performance over the individual methods. The consensus interface residue predictions are indicated by an "E" in Figure 1, and performance results are summarized in the last rows of Tables 1 and 2. The consensus method generally results in an enhanced correlation coefficient and sensitivity+, demonstrating the superior performance of the consensus method for identifying interface residues in this protein set. Predictions for each protein, provided in Supplementary Materials [see Additional files 1, 2, 3, 4, 5, and 6], illustrate that the improvements can be even more pronounced when the individual predictions of all four methods are relatively weak. This suggests that combining diverse prediction methods may be an excellent approach for the prediction of the binding sites in protein complexes.

## Conclusions

Each of the four prediction methods presented in this paper sheds a different light on the conservation and prediction of protein interaction sites, but none of the methods taken separately is as powerful as the combination of all four methods. The simple consensus approach presented here could perhaps be improved by generating an ensemble predictor with more detailed probabilities. Our current work is directed at this approach. It is clear that the present subject is an active field of research [31-38].

## Methods

### Dataset of hydrolase-inhibitor complexes

The dataset of 7 hydrolase-inhibitor complexes used in this work has been derived from a larger dataset of 70 protein heterocomplexes extracted from PDB by Chakrabarti and Janin [39] and used in our previous studies [2,3]. All are proteins from hydrolase-inhibitor complexes, with six being proteinases: 1acb_E [40] (chain E of PDB structure 1acb), 1fle_E[41], 1hia_A[42], 1avw_A[43], 2sic_E[44], 3sgb_E [18]; and one being a carboxypeptidase: 4cpa [45].

### Definition of surface and interface residues

Surface and interface residues for the proteins were identified based on information in the PDB coordinate files as previously described [2,3]. Briefly, solvent accessible surface areas (ASA) for each residue in the unbound protein and in the complex are calculated using DSSP [26]. A surface residue is defined as an interface residue if its calculated ASA in the complex is less than that in the monomer by at least 1 Å^2 ^[46]. In the extraction of interfacial region for threading, however, a distance-based definition of surface is used: a surface residue is defined as an interface residue if its side-chain center is within 6.5Å of the side-chain center of a residue belonging to another chain in the complex.

Based on the ASA definitions, 41% of the residues in the set of 7 proteins were surface residues, corresponding to a total of 631 surface residues. Among these surface residues, 166 were defined as interface residues and 465 as non-interface residues (i.e. surface residues that are not in the interaction sites). Thus, on average, interface residues represent 26% of surface residues, or 11% of total residues for proteins in our dataset.

### Using phylogeny to identify conserved residues

Many computational tools have been developed for identifying amino acids that are important for protein function/structure, but there is no consensus regarding the best measure for evolutionary conservation [47]. Evolutionary conservation can be decomposed into three components: i) the overall selective constraints – the number of changes observed at a site; ii) the pattern of amino acid substitutions – the number of amino acid types observed at a site; and iii) the effect of amino acid usage. We have established a reliable relationship between each measure and various aspects of structure. To explore the connection between sequence conservation and functional-structural importance, we proposed a new measure that can decompose the conservation into these three components [47]. This measure is based on phylogenetic analysis. The evolutionary rate at site *k *during lineage *l *from amino acids *i *to *j *(*i*,*j *= 1,...20) can be expressed as λ_*kl *_(*i*,*j*) = *c*_*k *_× *a*_*lk *_× *Q*(*i*,*j*|*k*), where *c*_*k *_accounts for the rate variation among sites, *a*_*lk *_for site-specific lineage (or subtree) effect caused by functional divergence [48], and the 20 × 20 matrix *Q*(*i*,*j*|*k*) is the (site-specific) model for amino acid substitutions. The likelihood function for a given tree can be determined according to a Markov chain model [49]. We have developed an integrated computer program (DIVERGE [50]) that can map these predicted sites onto the protein surface to examine these relationships. We use the solvent accessibility data from DSSP [26] to restrict predicted conserved residues to those located on the protein surface.

### Conservatism of conservatism

The phylogeny-based conservation of residues relies on sequence homology. It is well known, however, that many non-homologous proteins share similar folds [51]. It is therefore highly desirable to study the conservation of residues in proteins based on the structural superimposition of non-homologous proteins. In order to obtain insight into the evolutionary conservation of residues in proteins, we use the Conservatism of Conservatism method (CoC). The CoC method was developed by Mirny and Shakhnovich [21] for studying evolutionary conservation of residues in proteins with specific folds from the FSSP database [23]. With the FSSP database, Mirny and Shakhnovich performed an analysis of conserved residues in several common folds. The 20 naturally occurring amino acids were subdivided into 6 different classes, based on their physicochemical characteristics and frequencies of occurrence at different positions in multiple sequence alignments. The evolutionary conservatism within families of homologous proteins was measured through sequence entropy. Structural superimposition of different families of proteins with similar folds was used to calculate CoC for all positions of residues within a fold. Here we have applied a similar approach to identify structurally conserved residues involved in protein interactions.

For each protein, we first calculate the sequence entropy at each position within a family of related sequences from the HSSP database [25]



where  is the frequency of the class *i *of residues (for each of the six classes) at position *l *in sequence in the multiple sequence alignment. Then we use the FSSP database to obtain the structural alignment. The structural superimposition of different families was used to calculate the conservatism of conservatism (CoC)



where *s*^*m*^*(l) *is the intrafamily conservatism within the family *m *at position *l*, and *M *is the number of families. The CoC is the measure of the evolutionary conservation of the specific sites within the protein fold. Because the CoC method does not distinguish between residues at the protein surface evolutionarily conserved for functional reasons and residues inside the protein core that are conserved because of their importance to the folding process, we use solvent accessibility data for the unbound molecules to eliminate those conserved residues located inside the protein core.

### Data mining approaches to binding site identification

Recent advances in machine learning [52] or data mining [53] offer a valuable approach to the data-driven discovery of complex relationships in computational biology [54,55]. In essence, a data mining approach uses a *representative data training set *to extract complex *a priori *unknown relationships, e.g., sequence correlates of protein-protein interactions. Examination of the resulting classifiers can help generate specific hypotheses that can be pursued using molecular and biophysical methods. For example, a classifier that is able to identify protein-protein interface residues on the basis of sequence or structural features can provide insights about sequence characteristics that contribute to important differences in function. The data mining approach for binding site identification consists of the following steps:

• Identify the surface residues in each protein.

• Label each residue in each protein as either an interface residue or a non-interface residue based on appropriate criteria for defining residues in interaction sites.

• Use a machine learning algorithm to train and evaluate a classifier to categorize a target amino acid as either an interface or a non-interface residue. Different types of information about the target residue (e.g., the identity and physicochemical properties of its sequence neighbors, whether or not the target residue is a surface residue) can be supplied as input to the classifier. A variety of machine learning algorithms [52,54] can be used for this purpose.

• Evaluate the classifier (typically using cross-validation or leave-one-out experiments) on independent test data (not used to train the classifier).

• Apply the classifier to identify putative interface residues in a protein, given its sequence (and possibly its structure), but not the sequence or structure of its interaction partner.

Here we have used a support vector machine (SVM) learning algorithm because SVMs are well-suited for the data-driven construction of high-dimensional patterns and are especially useful when the input is a real-valued pattern [56]. In addition, algorithms for constructing SVM classifiers effectively incorporate methods to avoid over-fitting the training data, thereby improving its generality, i.e., the performance of the resulting classifiers on test data. Support vector machine algorithms have proven effective in many applications, including text classification [57], gene expression analysis using microarray data [58], and predicting whether or not a pair of proteins is likely to interact [59].

### Threading of sequences through structures of protein-protein interface surfaces

In phylogenetic and data mining approaches, the properties of the protein-protein interface are deduced by concentrating on the sequence information contained in the protein pair under investigation. However, it is well accepted that the physical origin of the specificity of protein-protein interactions comes predominantly from their structures. Thus, in any thorough investigation of protein-protein interactions, it is essential to include information from structural studies. Here we have adapted methods employed in protein structure recognition [60-63] to the problem of predicting protein-protein interface residues. In the first stage, structural models for identifying protein-protein interfaces are generated from existing protein databank (PDB) structures by extracting portions of contacts between different protein chains. We found that if we define the interaction region by the criterion that backbone C^α ^atoms on the two interacting chains are less than 15 Å apart, reasonably well connected fragments suitable for threading studies are obtained. In the second stage, after identifying a set of candidate template structures, threading is performed to examine the probability that a given model resembles the real interface. The threading algorithm is described in Cao et al. [27]. The threading alignments and scores obtained allowed us to predict which parts of each protein are in the interfacial region in the hydrolase-inhibitor complexes and to predict the most probable residue-residue contacts between the two proteins.

### Ensemble predictions for combining results from multiple methods

Different approaches for identifying binding sites from amino acid sequence information yield different (sometimes contradictory, sometimes complementary) results. In such cases, approaches for combining results from multiple predictors have a potential importance. The key idea is that results obtained by using different approaches, which we will call classifiers henceforth, may be correlated (or, more generally, statistically dependent) due to a variety of reasons including the use of a common dataset for constructing or tuning classifiers, use of intermediate variables for encoding input to the classifiers, and similarities between methods (e.g., SVM, neural networks). Regardless of the source of statistical dependency, the goal is to develop methods for weighting the output of each classifier appropriately for the purpose of producing more accurate predictions. Our method takes as input the binary (True/False) output of each classifier (e.g., SVM, CoC) and produces as output a probability that the residue under consideration is an interface residue, using the outputs produced by each of the classifiers. Algorithms for learning Bayesian (or Markov networks) can be then used to learn the network of dependences and the relevant conditional probabilities.

### General evaluation measures for assessing the performance of classifiers

Let *TP *denote the number of true positives – residues predicted to be interface residues that are actually interface residues; *TN *the number of true negatives – residues predicted not to be interface residues that are in fact not interface residues; *FP *the number false positives – residues predicted to be interface residues that are not interface residues;*FN *the number of false negatives – residues predicted not to be interface residues that actually are interface residues. Let *N *= *TP*+*TN*+*FP*+*FN*. Sensitivity (recall) and Specificity (precision) are defined for the positive (+) class as well as the negative (-) class. Sensitivity^+ ^= *TP*/(*TP*+*FN*), Sensitivity^-^= *TN*/(*TN*+*FP*), Specificity^+ ^= *TP*/(*TP*+*FP*), Specificity^- ^=*TN*/(*TN*+*FN*). Overall sensitivity and overall specificity correspond to expected values of the corresponding measures averaged over both classes. The performance of the classifier is summarized by the correlation coefficient, which is given by



The correlation coefficient ranges from -1 to 1 and is a measure of how predictions correlate with the actual data [64]. It is important to note, that when the number of negative instances is much larger than the number of positive instances – as is the case for prediction of interface residues – the Sensitivity+ and Specificity+ measures are more appropriate for assessing prediction performance than the overall Sensitivity and Specificity measures [64]. In the extreme case when a classifier predicts every example to be negative (due to a preponderance of negative training instances) these overall performance measures would still show a high success rate despite the obvious failure of the prediction method. In such cases, the Correlation Coefficient, as well as the Sensitivity+, which is a measure of the fraction of positive instances that are correctly predicted, and Specificity+, which is a measure of the fraction of the positive predictions that are actually positive instances, may provide better performance assessment. Of course, a meaningful comparison of the performance of different classification methods depends critically on the specific application and goal.

## Author's contributions

CY, VH and DD performed data mining calculations. XG performed phylogenetic calculations. KMH, CZW, YI, DD, and HC worked on threading. TZS, AK, and RLJ worked on the implementation of CoC and the development of consensus methodology. Every author contributed to the final draft of the paper.

## Supplementary Material

Additional File 1 Comparison of individual methods for interface residue prediction for bovine α-chymotrypsin (1acbe). Click here for file

Additional File 2 Comparison of individual methods for interface residue prediction for porcine pancreatic trypsin (1avwa). Click here for file

Additional File 3 Comparison of individual methods for interface residue prediction for porcine pancreatic elastase (1flee). Click here for file

Additional File 4 Comparison of individual methods for interface residue prediction for kallikrein(1hiaa). Click here for file

Additional File 5 Comparison of individual methods for interface residue prediction for subtilisin BPN' (2sice). Click here for file

Additional File 6 Comparison of individual methods for interface residue prediction for carboxypeptidase A (4cpa). Click here for file
