# Supplementary material for: Predicting binding sites of hydrolase-inhibitor complexes by combining several methods
Source: BMC Bioinformatics. 2004 Dec 17;5:205. doi: 10.1186/1471-2105-5-205 (PMC544855; doi:10.1186/1471-2105-5-205)
Supplement: Additional File 1 — Comparison of individual methods for interface residue prediction for bovine α-chymotrypsin (1acbe). [file 1471-2105-5-205-S1.pdf]

```
        10        20        30        40        50        60
CGVPAIQPVLSGLIVNGEEAVPGSWPWQVSLQDKTGFHFCGGSLINENWVVTAAHCGVTT
                                       P              PP
                 CC  C            CC CCC     C C      CCC CC
        S           S             S SSSS              SS
                                    TTT               TTT
                                  E EEEE              EEE

        70        80        90       100       110       120
SDVVVAGEFDQGSSSEKIQKLKIAKVFKNSKYNSLTINNDITLLKLSTAASFSQTVSAVC

C         C   C    C   CCC C C  C  CC       C  CC  CC
                 S       S        S S              S
                                    T
                         E          E              E

       130       140       150       160       170       180
LPSASDDFAAGTTCVTTGWGLTRYXXANTPDRLQQASLPLLSNTNCKKYWGTKIKDAMIC

C   CC    CC      C                      C       C  C
  S               S                         S   SS
                    T  T  T                      T  T
                  E                             EE

       190       200       210       220       230       240
AGASGVSSCMGDSGGPLVCKKNGAWTLVGIVSWGSSTCSTSTPGVYARVTALVNWVQQTL
        P P P                    P
  CCC  CCCC         CC        CCCC CCC     C         C   C
  S    SSS  S        S        S S    S
       TTTT T                 T TTTT T     T
  E    EEEE E        E        E EE E E     E

       250
AAN
```

Rows :
1. Phylogeny (P)
2. COC (C)
3. SVM (S)
4. Threading (T)
5. Consensus (E)

Protein: 1ACB_E
