# Supplementary material for: Predicting binding sites of hydrolase-inhibitor complexes by combining several methods
Source: BMC Bioinformatics. 2004 Dec 17;5:205. doi: 10.1186/1471-2105-5-205 (PMC544855; doi:10.1186/1471-2105-5-205)
Supplement: Additional File 2 — Comparison of individual methods for interface residue prediction for porcine pancreatic trypsin (1avwa). [file 1471-2105-5-205-S2.pdf]

```
        10        20        30        40        50        60
IVGGYTCAANSIPYQVSLNXXSGSHFCGGSLINSQWVVSAAHCYKSRIQVRLXGEHNIDV
                          P              PP
    CC  C C              CC     C C      CCC CC          C
    SS    S              SS              SSSSS           S S
                       T TT              TT T
    EE    E              EE              EEEEE           E

        70        80        90       100       110       120
LEGNEQFINAAKIITHPNFNGNTLDNDIMLIKLSSPATLNSRVATVSLPRXSCAAXAGTE
                          P
 C    C   CC CC C CC  CC  C       CC  CC    C
S     S      S  S S    S              S
                  T TT T  T
      E      E  E E    E  E           E

       130       140       150       160       170       180
CLISGWGNTKSSGSSYPSLLQCLKAPVLSDSSCKSSYPGQITGNMICVFLEGKDSCQGDS
                                                     P PPP P
        C C C  C            C            C       CC CCCCCC
                               S                 S    SS
             T T                       T             TTTTT T
               E                                 E   EEEEE

       190       200       210       220       230
GGPVVCNGQXXXXLQGIVSWGYGXCQKNKPGVYTKVCNYVNWIQQTIAAN
                 P            P
                 C            C   C  C  C  C
                 S                           S
                 T  T   T     T
                 E            E
```

Rows :
1. Phylogeny (P)
2. COC (C)
3. SVM (S)
4. Threading (T)
5. Consensus (E)

Protein: 1AVW_A
