# Supplementary material for: Predicting binding sites of hydrolase-inhibitor complexes by combining several methods
Source: BMC Bioinformatics. 2004 Dec 17;5:205. doi: 10.1186/1471-2105-5-205 (PMC544855; doi:10.1186/1471-2105-5-205)
Supplement: Additional File 3 — Comparison of individual methods for interface residue prediction for porcine pancreatic elastase (1flee). [file 1471-2105-5-205-S3.pdf]

```
         10        20        30        40        50        60
VVGGTEAQRNSWPSQISLQYRSWAHTCGGTLIRQNWVMTAAHCVDRELTFVVVGEHNLNQ
          P        P     PP              PP
    CC  CCC          CC  CC     C        CC  CCCC
                      S  SS      S       SS  S S
                         TT               T
          E           E  EE              EE  E E

         70        80        90       100       110       120
NNGTEQYVGVQKIVVHPYWNTDDVGYDIALLRLAQSVTLNSYVQLGVLPRAGTILANNSP
                                               P   P
      C   CCC C C  C  CC C     C  CC   CC   C CC     C   C
      S     S S S        S            S  S  S SS     S     S
                       T
      E     E E EE     E E                  E EE     E

        130       140       150       160       170       180
CYITGWGLTRXTNGQLAQTLQQAYLPTVDYAICSSYWGSTVKNSMVCAGGDGVSGCQGDS
 P                          P       P P                P P P
               C            C       C    C        C C  CCC
 S                           S S    S                  S
       T                            T  T              T T  T
 E                          E       E                  EEE

        190       200       210       220       230
GGPLHCLVNGQYAVHGVTSFVSLGCNTRKPTVFTRVSAYISWINNVIASN
         P        PP P   P  P
         CC      CCCC    C              C
      SS  SS     S SS S  S
                 T TT T
         EE      EEEE E  E
```

Rows :
1. Phylogeny (P)
2. COC (C)
3. SVM (S)
4. Threading (T)
5. Consensus (E)

Protein: 1FLE_E
