# Supplementary material for: Predicting binding sites of hydrolase-inhibitor complexes by combining several methods
Source: BMC Bioinformatics. 2004 Dec 17;5:205. doi: 10.1186/1471-2105-5-205 (PMC544855; doi:10.1186/1471-2105-5-205)
Supplement: Additional File 4 — Comparison of individual methods for interface residue prediction for kallikrein(1hiaa). [file 1471-2105-5-205-S4.pdf]

```
        10        20        30        40        50        60
IIGGRECEKNSHPWQVAIYHYXSSFQCGGVLVNPKWVLTAAHCKNDNYEVWLXGRHNLFE
                          P              PP
    CC CCCC               C              CCCCC
    S   SSS               S              SS S S           S
                      TT TT              TT
    E   EEE               E              EE E

        70        80
NENTAQFFGVTADFPHPGFN

          C C
S     S
```

Rows :
1. Phylogeny (P)
2. COC (C)
3. SVM (S)
4. Threading (T)
5. Consensus (E)

Protein: 1HIA_A
