# Supplementary material for: Predicting binding sites of hydrolase-inhibitor complexes by combining several methods
Source: BMC Bioinformatics. 2004 Dec 17;5:205. doi: 10.1186/1471-2105-5-205 (PMC544855; doi:10.1186/1471-2105-5-205)
Supplement: Additional File 5 — Comparison of individual methods for interface residue prediction for subtilisin BPN' (2sice). [file 1471-2105-5-205-S5.pdf]

```
         10        20        30        40        50        60
AQSVPYGVSQIKAPALHSQGYTGSNVKAVIDSGIDSSHPDLKVAGGASMVPSETNPFQD
     P     P      PP    P P          P P      P       P
     C                  C C
                  SS      S          S S  S           SS

     E            EE    E E          E E              E

         70        80        90       100       110       120
NNSHGTHVAGTVAALNNSIGVLGVAPSASLYAVKVLGADGSGQYSWIINGIEWAIANNMD
   P          P P         P        P   PPP PP P    P    P  P
CC C                      C C      C   C CCCC C C  C   CCC C
SS S          S   S                S  S   S     S  S
 T T                               T  TTTTTT  T
EE E          E           E        E  EE EEEE E E  E    E  E

        130       140       150       160       170       180
VINMSLGGPSGSAALKAAVDKAVASGVVVVAAAGNEGTSGSSSTVGYPGKYPSVIAVGAV
    PPPPP PP P P   P     P     P  P   PP      PP
    CCCC C   CCCC  CC CCCC     C  C   CCC     CC   C
    SS                         S  SS          SS
     TTTTT    T                T TTT           T
    EEEE E   EEE   E     E     E  EE  EE      EE

        190       200       210       220       230       240
DSSNQRASFSSVGPELDVMAPGVSIQSTLPGNKYGAYNGTSMASPHVAGAAALILSKHPN
 P     PP     P                  P   PPPPP                P
 CCCC  CC    C         C C     CCC CCCCCCC                C
    S  S     S                   S    S
       TT                            T  TT
 E  E  EE    E                   E  EEEEEE                E

        250       260       270
WTNTQVRSSLENTTTKLGDSFYYGKGLINVQAAAQ
    P       P P PP P P  P     P
 C C       CC C C
 S SS      S    SS S

 E EE      EE E EE E
```

Rows :
1. Phylogeny (P)
2. COC (C)
3. SVM (S)
4. Threading (T)
5. Consensus (E)

Protein: 2SIC_E
