# Supplementary material for: Predicting binding sites of hydrolase-inhibitor complexes by combining several methods
Source: BMC Bioinformatics. 2004 Dec 17;5:205. doi: 10.1186/1471-2105-5-205 (PMC544855; doi:10.1186/1471-2105-5-205)
Supplement: Additional File 6 — Comparison of individual methods for interface residue prediction for carboxypeptidase A (4cpa). [file 1471-2105-5-205-S6.pdf]

```
        10        20        30        40        50        60
ARSTNTFNYATYHTLDEIYDFMDLLVAQHPELVSKLQIGRSYEGRPIYVLKFSTGGSNRP
                      P                     P     P        P
       C C   C  C           CC   CCC   C CCCC     C  CCC C C
     S                                      S         S S

                                            E     E   E    E

        70        80        90       100       110       120
AIWIDLGIHSREWITQATGVWFAKKFTENYGQNPSFTAILDSMDIFLEIVTNPNGFAFTH
        P PP
        C CC                 C  C       C                  C
        S                            S   S                 S
           T
        E EE                                               E

       130       140       150       160       170       180
SENRLWRKTRSVTSSSLCVGVDANRNWDAGFGKAGASSSPCSETYHGKYANSEVEVKSIV
   P                   PP               PP
C C C C        C       CC             C CCCC         C  C
S     S       SS        S  S   SS        S
    T                   T                 TT
E   E E        E       EE               EEEE

       190       200       210       220       230       240
DFVKNHGNFKAFLSIHSYSQLLLYPYGYTTQSIPDKTELNQVAKSAVAALKSLYGTSYKY
               P PP                                   P
C     C  C     CCCC C C       C   CC               CCCC C CC
S  S     S      S                  SS  S   S               S
                 TT T
E        E     EEEE E              E                  E    E

       250       260       270       280       290       300
GSIITTIYQASGGSIDWSYNQGIKYSFTFELRDTGRYGFLLPASQIIPTAQETWLGVLTI
  P    P P             P     P        P  PP
  CC CCCCC         CCC C   C C    CCC CC C C          C
      S            SSS             S   S S        S   S
     TTT                     T        T
  E  EEE E         EEE E     E     E  EE E            E

       310
MEHTVNN
```

<u>Rows</u> :
1. Phylogeny (P)
2. COC (C)
3. SVM (S)
4. Threading (T)
5. Consensus (E)

Protein: 4CPA
